# COVID-19 vaccination readiness among multiple racial and ethnic groups in the San Francisco Bay Area: A qualitative analysis

**DOI:** 10.1371/journal.pone.0266397

**Published:** 2022-05-12

**Authors:** Jonathan Z. Butler, Mariam Carson, Francine Rios-Fetchko, Roberto Vargas, Abby Cabrera, Angela Gallegos-Castillo, Monique LeSarre, Michael Liao, Kent Woo, Randi Ellis, Kirsten Liu, Arun Burra, Mario Ramirez, Brittney Doyle, Lydia Leung, Alicia Fernandez, Kevin Grumbach

**Affiliations:** 1 Department of Family and Community Medicine, University of California, San Francisco, CA, United States of America; 2 Department of Medicine, UCSF LatinX Center for Excellence, University of California, San Francisco, CA, United States of America; 3 Instituto Familiar de la Raza, Inc., San Francisco, CA, United States of America; 4 Rafiki Coalition, San Francisco, CA, United States of America; 5 NICOS Chinese Health Coalition, San Francisco, CA, United States of America; 6 Stanford University, Stanford, CA, United States of America; 7 Department of Medicine, UCSF Black Health Initiative, University of California, San Francisco, CA, United States of America; LSHTM, UNITED KINGDOM

## Abstract

**Background:**

COVID-19 vaccination rates are lower among historically marginalized populations, including Black/African American and Latinx populations, threatening to contribute to already high COVID-19 morbidity and mortality disparities for these groups. We conducted a community-based participatory research study using qualitative methods to explore knowledge and beliefs about COVID-19 vaccination among Black/African American, Latinx, and Chinese American residents of the San Francisco Bay Area and assess their views on vaccination outreach and delivery strategies.

**Methods and findings:**

Data were collected from January 14, 2021, to February 24, 2021, with adult residents (N = 109 [Female: N = 76; 70%]) in San Francisco. Focus groups (N = 10) and in-depth interviews (N = 25) were conducted among Black/African Americans (N = 35), Latinx (N = 40), and Chinese Americans (n = 34) in English, Spanish, Cantonese, or Mandarin. Themes were identified using grounded field theory, and included misinformation, mistrust of government and health institutions, and linguistic and other barriers to vaccine access. All three racial/ethnic groups had experiences with vaccine misinformation and information overload. Many African American and Latinx participants cited structural and interpersonal racism, and anti-immigrant discrimination, as factors reducing their trust in government and public health disseminated information and their willingness to be vaccinated. Participants expressed trust in community-based organizations, including faith-based organizations and community-run clinics. Participants often experienced barriers to vaccine access, such as transportation to drive-in sites, with Latinx and Chinese American groups also frequently citing language barriers.

**Conclusions:**

Vaccine outreach strategies must acknowledge how longstanding systemic, institutional, and structural racism contributes to mistrust in government and health institutions and engage with and support trusted messengers from the community to eliminate cultural, linguistic, and other barriers to vaccine access.

## Introduction

COVID-19 has had a disproportionate toll on historically marginalized populations in the United States. During the first year of the pandemic, the number of excess deaths in the US relative to mortality rates in 2015–19 were 54% higher among Latinx, 37% higher among Asian, 33% higher among Black, and 29% higher among American Indian and Alaska Native populations, in comparison to 12% higher among the white population [1]. These inequities were largely an outcome of structural racism and related systemic factors, such as crowded housing, incarceration, and employment in low paying job as an essential front-line worker. These inequities were largely an outcome of structural racism and related systemic factors, such as crowded housing, incarceration, and employment in low paying job as an essential front-line worker [2–5].

Vaccination is the cornerstone of preventing COVID-19 and its severe complications. However, at the time this study was initiated in the winter of 2020, there was concern that disparities in vaccination access and uptake would exacerbate the racial-ethnic inequities in COVID-19 morbidity and mortality. Surveys conducted prior to emergency use authorization of COVID-19 vaccines in the US indicated that communities of color had lower confidence in COVID vaccines than white individuals [6]. Racial-ethnic disparities became evident early in vaccine rollout. In March, 2021, 13% of white, 11% of Asian, 7% of Latinx, and 5% of Black people had received at least one dose of a COVID-19 vaccine [7]. Although vaccination rates rapidly increased among all groups in the spring of 2021, with Asian populations achieving the highest rate (82%) by May 2021 among adults aged 18 years or older, vaccination rates among other populations of color continued to lag behind that of the white population; the percentage of adults with at least one vaccine dose by May 2021 was 68% for white, 40% for American Indian, 59% for Black, 55% for Latinx, and 40% for Native Hawaiian and Pacific Islander populations [8].

Vaccination requires both individual and health system readiness. The conceptual framework of readiness has been proposed as an alternative to the often-used framework of vaccine hesitancy [9], in response to criticisms that the hesitancy framework unduly blames the individual for inequities that are largely manifestations of systemic problems [10]. The readiness framework recognizes both the role of the individual in making an informed decision about readiness for vaccination, and the role of the health system in dismantling access barriers to vaccination and proactively addressing the needs of socially marginalized groups. Most research on COVID-19 vaccine disparities has focused on individual readiness. A recent review of published national surveys in the US identified several factors significantly associated with lower individual vaccine readiness among Latinx and Black/African American individuals, including socioeconomic status, medical mistrust, racial discrimination, exposure to misinformation and myths, and mistaken beliefs about vaccine efficacy and potential side effects [11]. Specifically, low vaccination readiness was attributed to those who were younger in age, have lower income and no college degree, have conversative political leanings, living in rural neighborhoods, living in multigenerational households, mistrust medical systems and have experienced racial discrimination [12–17]. Some surveys in the US have also found less vaccine confidence among Asian individuals relative to white respondents [12]. Surveys in the United Kingdom have revealed similar differences between white and minoritized populations in their beliefs about COVID-19 vaccination [18]. Most published research comparing racial and ethnic groups’ perspectives about COVID vaccination has used quantitative survey methods that have limited ability to explore nuance in knowledge, beliefs, and experiences. Additionally, research has focused more on individual readiness than on accessibility of vaccination and other aspects of health system vaccination readiness.

Published qualitative analyses on COVID-19 vaccination perspectives show that Black and Latinx individuals generally have lower individual readiness to vaccination than white individuals. However, attitudes are on a continuum from people who were strongly for or firmly against receiving the COVID-19 vaccinations [19]. Qualitative studies have established that barriers to vaccination are medical and governmental mistrust, inadequate information provided about the vaccination, concerns with an accelerated timeline, and limited data on long-term effects; facilitators to vaccination include informing trust via trusted messengers, community-led advocacy, addressing structural barriers to access and technology, and physician-delivered messages [19–29]. Although several qualitative analyses on COVID-19 vaccination perspectives focus on one group, or some combination of Black and Latinx communities, limited studies include perspectives from Chinese ethnic communities, especially in the US [27].

We conducted a community-based participatory research study using qualitative methods to explore knowledge and beliefs about COVID-19 vaccination among Black/African American, Latinx, and Chinese American residents of the San Francisco Bay Area and assess their views on vaccination outreach and delivery strategies. These three racial-ethnic groups are the most populous non-white groups in the region. The study was conducted coincident with the first emergency use authorizations of COVID-19 vaccines in the US (figure) when concerns about racial-ethnic inequities in vaccination were emerging. The study’s overarching goal was to incorporate a diverse set of perspectives from historically marginalized populations into public health vaccination outreach and delivery recommendations to eliminate vaccination inequity.

## Methods

**Fig 1:**
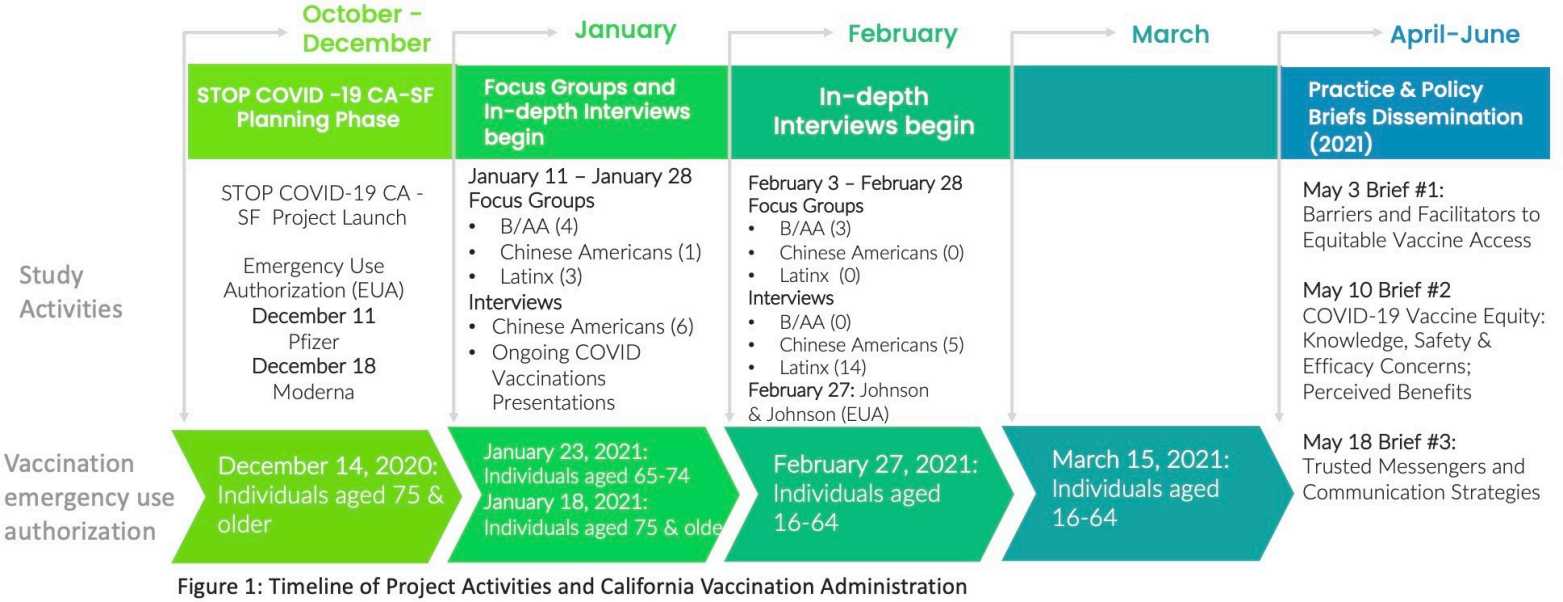
Timeline of project activities and California Vaccination administration.

We conducted focus groups and in-depth interviews from January 14, 2021, to February 24, 2021, with adult San Francisco Bay Area residents. This period overlapped with the California Department of Public Health authorizing vaccination administration to persons in the general population aged 75 years and older (January 18, 2021), persons 65–74 years old (January 23, 2021), emergency services workers (February 27, 2021), and persons 16–64 years old with comorbidities (March 15, 2021) (Fig 1). Vaccinations for healthcare workers were authorized in December 2020, before our study. Very few study participants were eligible to be vaccinated when they participated in the study, and the vaccine supply was limited for the eligible few.

### Community-based organization and university collaboration

We employed community-based participatory research principles [30,31] in partnership with three community-based organizations (CBOs) serving the priority populations included in the study: Instituto Familiar de la Raza; NICOS Chinese Health Coalition; and the Rafiki Coalition. All are longstanding organizations in San Francisco that have previously collaborated to improve health for socially marginalized populations in San Francisco and partnered with UCSF team members on public health and community based participatory research projects. Instituto Familiar de la Raza promotes the health and well-being of the Chicano/Latino/Indigena community of San Francisco through a wide range of programming including HIV, violence, trauma, or other behavioral & mental health issues. NICOS Chinese Health Coalition’s mission is to enhance the health and well-being of San Francisco’s Chinese community through advocacy, research, training, and coalition building, through networking of Asian-serving organizations. The Rafiki Coalition focuses on reducing health inequities that impact the Black/African American community through health education, individual and family therapy, nutrition workshops and health screenings, and other programs. CBO and university staff collaborated in all facets of the project, including focus group and interview guide development, participant recruitment, conducting focus groups and interviews, data analysis and interpretation, and writing and dissemination of study products. The university team members included faculty and staff racially-ethnically and linguistically concordant with the groups studied, with well-established relationships with the communities engaged. The study was approved by the UCSF Institutional Review Board (study #20–32672).

### Participant recruitment

CBO staff used phone banking, emails, and printed fliers to invite eligible clients and other constituents of their community networks to participate in the study. Eligible participants were persons 18 years of age or older who identified as either Black/African American, Latinx, or Chinese American and spoke English, Spanish, Mandarin or Cantonese. Following initial recruitment, university staff members screened individuals for eligibility and obtained informed consent. Individuals preferring an individual interview rather than focus group participants were interviewed separately. CBOs offered technical support to accommodate participants unfamiliar with videoconferencing. Focus groups and interviews were conducted remotely through Zoom or by phone. They were recorded with permission, transcribed in the language used, and then translated to English for the Spanish and Chinese transcripts. Focus group participants and interviewees received a $100 and $50 check, respectively.

### Focus group and interview procedures

Immediately preceding each focus group or interview, participants completed a short questionnaire with demographics, occupational status, household size, and COVID-19 status items. Focus groups and interviews were conducted in English, Spanish, or Cantonese depending on participant-preferred language and co-facilitated by language and culturally concordant university and CBO staff members. Nine CBO and university staff conducted the interviews and focus groups (Black/African American (2), Latinx (5) and Asian (2)). Focus groups and interviews began with questions about general COVID-19 experiences and attitudes. Open-ended questions explored knowledge and beliefs about COVID-19 vaccination (S1 Table). After each focus group, participants were invited to remain for a ten-minute COVID-19 vaccination educational presentation from one of the facilitators, followed by a question-and-answer discussion.

### Data analysis

Utilizing a modified grounded theory, two members of the research team read a subsample of transcripts to identify emerging themes [32]. These themes were then discussed with the entire research team, including CBO representatives. Using the themes from the sampled transcripts, the entire research team developed a codebook through a card sorting exercise facilitated by a virtual whiteboard. The study team used this approach due to the participatory nature of the analysis benefiting from a more visual tool with CBO partners. Discrepancies in codes were sorted through group discussion and consensus. This codebook was applied to the remaining transcripts by the same two team members that read the first subsample of transcripts. The codebook was iteratively refined as more transcripts were coded.

## Results

One hundred nine individuals participated in the study, 84 in 10 focus groups and 25 in individual interviews. Participant characteristics are shown in Table 1 and key themes in Table 2.

**Table 1:** Demographic characteristics by race ethnicity (N = 109).

Characteristic, N, (%)	Overall	Black/African American	Latinx	Chinese American
	N = 109	N = 35 (32.1)	N = 40 (36.7)	N = 34 (31.2)
**Age** 18–40 41–64 65+	38 (34.8) 56 (51.4) 15 (13.8)	11 (28.9) 15 (26.8) 9, (60)	12 (31.6) 24 (42.9) 4 (26.7)	15 (39.5) 17 (30.3) 2 (13.3)
**Sex** Female Male	76 (69.7) 33 (30.3)	22 (62.9) 13 37.1)	24 (60.0) 16 (40.0)	30 (88.2) 4 (11.8)
**Interview type** Individual interviews Focus Groups	25 (22.9) 84 (77.0)	0 (0) 35 (100)	14 (56) 26 (44)	11, (44) 23, (56)
**Work outside the home** Yes No	45 (41.3) 64 (58.7)	24 (53.3) 11 (17.2)	22 (48.9) 12 (18.8)	11, (24.4) 22, (34.4)
**Preferred Language** English Cantonese Mandarin Spanish	41 (37.6) 30 (27.5) 3 (2.8) 35 (32.1)	35 (100.0) 0 (0) 0 (0) 0 (0)	5 (12.5) 0 (0) 0 (0) 35 (87.5)	1 (3.0) 30 (88.2) 3 (8.8) 0 (0)
**Household size** 1 2–3 4–5 6+	21 (19.3) 32 (29.3) 47 (43.1) 8 (7.3)	13 (37.1) 11 (31.4) 10 (28.6) 0 (0)	6 (15) 15 (37.5) 16 (40.0) 3 (7.5)	2 (5.8) 6 (17.6) 21 (61.8) 5 (14.7)
Household members over 64* 0 1 2	95 (87.2) 10 (9.2) 4 (3.6)	33 (34.7) 2 (20) 0 (0)	37 (92.5) 3 (7.5) 0 (0)	25 (73.5) 5 (14.7) 4 (11.8)
**Household members under 18** 0 1–2 3+	54 (49.5) 48 (44.0) 7 (6.4)	28 (80.0) 6 (17.0) 1 (2.9)	22 (55.0) 15 (37.5) 3 (7.5)	4 (11.8) 27 (79.4) 3 (8.8)
**Household members with medical conditions** Yes No	24 (22.0) 85 (78.0)	12 (34.3) 23 (65.7)	11 (27.5) 29 (72.5)	1 (2.9) 33 (97.1)
**Received flu shot in 2019 or 2020** Yes No	65 (59.6) 44 (40.4)	15 (42.9) 20 (57.1)	24 (60.0) 16 (40.0)	26 (76.5) 8 (23.5)
**Received COVID test** Yes No	69 (63.3) 40 (37.7)	28 (80.0) 7 (20.0)	31 (77.5) 9 (22.5)	10 (29.4) 24 (70.6)

Notes

* not including the age of the participant.

**Table 2:** Key themes.

**Knowledge and Beliefs**	
**Vaccine information challenges and misinformation**	Information overload and a need for easy to understand, concise information in preferred language, without technical jargon
	Rampant misinformation disseminated on social media and other sources
**Mistrust of government and health institutions**	Mistrust rooted in systemic racism and hostility to immigrants
	A belief that drug companies and the government are more interested in profit than community health
	Personal negative experiences with the healthcare system
**Perceived benefits of vaccination**	Economic, social, and health benefits as motivation for vaccination
	Loss of loved ones and community members and the need to prevent further loss of life
**Trusted Messengers**	
**Trust in community-based organizations**	Community-based organizations, including faith-based organizations and community-run clinics as part of community-based organizations
**Trust in healthcare providers**	Personal healthcare providers
**Vaccination Access**	
**Access barriers**	Transportation barriers causing frustration with drive-in only vaccination sites
	Language barriers, especially for Latinx and Chinese American participants with limited English proficiency
**Access facilitators**	Community-run vaccination locations
	Outreach services to help schedule appointments and provide vaccine information
	Language-concordant patient support for individuals with limited English proficiency

### Knowledge and beliefs

#### Vaccine information and misinformation

Many individuals in all three racial/ethnic groups had concerns about the vaccine’s safety and efficacy stemming from challenges in accessing and sorting through information, and confusion about ongoing scientific uncertainties, as well as due to receiving misinformation about vaccines. By misinformation, we mean messaging that grossly deviates from factual scientific evidence. Concerns about the vaccine included how well the vaccine protects against variants of the virus, what the long-term effects may be, how individuals with specific medical conditions may react to the virus, that production and safety testing were rushed, and that the vaccine may only protect for a short period.

*Information challenges*. Participants commented about an overload of information—presenting a challenge for individuals looking for trustworthy, evidence-based information—and a need for more concise information in their preferred language that is without technical jargon and is easy to understand.

*You can’t just put things out on people and say*, *this is great for you*, *and assume they’re always going to agree with you*. *That wouldn’t be human nature*. *It just needs to be more clear*, *more concise*, *and delivered in a way that little kids could even understand*. Female, African American

*Misinformation*. All three racial/ethnic groups had experiences with misinformation that undermined confidence in COVID-19 vaccines. Misinformation commonly mentioned by participants included the vaccine causing infertility, individuals dying from receiving the vaccine, the vaccine causing changes in DNA, the vaccine containing a microchip, people needing to pay to get the vaccine, and the vaccine not being available for those without legal documentation.

*A lot of people who don’t read books, they go onto YouTube, and that’s how they get informed, and there’s a lot of misleading information out there that talks about COVID and say, look, this isn’t real, or if you get the test, they’re putting micro-antibodies in you and all this different stuff that I’ve heard people say and even talk about it*.

Male, African American

Sources of misinformation included social media, news outlets, and friends and family members. Participants acknowledged that despite knowing social media disseminated misinformation, they still used it as their main source of information:

*I get my information*, *which is probably a terrible thing*, *from social media*. *I advise you not to trust social media*. *But even just like scrolling through my timelines*, *I just see a lot of negative comments about the vaccine*. *I think I saw a post*, *even when a nurse took it*, *and she apparently fainted*. *So*, *it was like a lot of distrust*. Male, African American

Immigrant participants recounted experiences arguing with family members and friends about COVID-19 misinformation circulating in their countries of origin:

*In my [home] country*, *multiple people in my family have died*. *My grandma*, *my aunt*, *my uncle*, *and there are some people that don’t believe in this disease and say*: *“no [COVID-19] is a lie; it doesn’t exist*.*” I say to my friends*, *“use hand sanitizer; go out with a mask*.*” “No*, *[COVID-19] doesn’t exist; it’s a lie*,*” they say*. *“It does exist because people in my family have died*,*” I tell them*. Female, Latinx

The challenges of making sense of information and filtering misinformation led many participants to adopt a “wait and see” approach.

*Because this vaccine was launched earlier than other vaccines and is not very mature*, *I am worried about adverse reactions*. *In two months*, *after many other people get vaccinated*, *I’ll see if there is any specific reaction or if the vaccine is improved*, *or if other pharmaceutical companies also release vaccines*. *When there are not so many adverse reactions*, *I will consider getting vaccinated*. Female, Chinese American

#### Mistrust

Participants from all three racial/ethnic groups frequently mentioned mistrust of governmental, medical, and other institutions. Participants attributed their mistrust of the vaccine to historical racial injustices, a belief that drug companies and the government are more interested in profit than community health, and negative personal experiences with the healthcare system. One statement from a Black/African American participant exemplified these sentiments:

*This mistrust we have in all the systems*, *all the powers that be*, *they haven’t historically treated Black people well in any system*, *whether it be medical*, *governmental*, *anything*, *so why start now*? Female, African American

Another participant expressed concern about how she would be taken care of if she had an adverse reaction to the vaccine:

*What if something happens with this vaccine*? *How will I then be treated*? *There’s a lot of issues with the fact of how we’re treated in the healthcare system if I were to get sick*. Female, African American

Although references to systemic racism as drivers of mistrust were most frequently mentioned in the Black/African American groups, participants in both the Black/African American and Latinx groups discussed feelings of governmental disregard toward their specific communities.

One participant expressed their concern around the timing of the vaccine and the government’s sudden interest in promoting uptake within the African American community:

*It’s like*, *why us*? *Why now*? *Why are you suddenly wanting black people to get this vaccine*? *You know what I mean*? *You kept everything from us; now*, *suddenly*, *you want us first in line for this vaccine that happened very quickly*. Female, African American

In the Latinx community, sentiments were quite similar; however, participants in this group tied their mistrust of the government to anti-immigrant sentiments.

*The Latinx community*, *the majority have a lot of fear in what they say*: *“They deny us everything*, *right*? *We don’t have health insurance; we don’t have funds for anything; we don’t count for anything because we are illegal*, *because we are immigrants*, *or whatever it may be*. *Why now are they offering us this*? *Like*, *it’s too good to be true*, *they want to use us*. Female, Latinx

The fractious, polarized 2020 presidential election campaign also contributed to eroding faith in government institutions.

*I don’t even trust the government*, *especially now with all this mess going on with Trump and Biden and all this*. *It’s like*, *really*, *who can we trust*? *I think it’s difficult to trust the government at this point because it’s just nasty*. Female, African American

Compared with Black/African American and Latinx participants, more of the Chinese American participants tended to regard government and health institutions as trustworthy. Chinese American participants mentioned that “the supervision of medical treatment and drugs is relatively strict [in the United States]” and that they had “confidence in the government and American medical care.”

Many participants across racial/ethnic groups expressed a need for acknowledgment of mistreatment and honest dialogue to work toward mending the broken relationships between medical and governmental institutions and communities of color.

*There needs to be a relationship-building piece before you can move forward*, *even if you want to educate*. *We have to lay down that groundwork to have the relationship*. *Because what moves people is the dialogue*. *Not the information*, *but you have to sit and have the dialogue*. Female, African American

#### Perceived benefits

When asked about their greatest motivators for getting a COVID-19 vaccine, participants’ answers fell into three main categories: economic; social; and health. Many participants were motivated to get the vaccine by the need for both personal and community economic recovery. Participants shared experiences and concerns of food and housing insecurity directly resulting from job loss due to the pandemic. As a result, one of the strongest motivators was the need to return to work safely. For example, Latinx community participants in the Spanish-speaking focus groups discussed their need to be vaccinated to engage in their service sector jobs, often at the request of employers. Participants also commented on the economic state of their communities as a motivator for uptake.

*I’m actually not afraid of taking the vaccine at all*. *I drove through downtown yesterday*, *and I’m noticing the economic impacts that are going to be so far-reaching as far as this is concerned that I’m willing to go ahead and sacrifice*. Female, African American

Participants discussed a yearning to return to social normalcy as a motivation to receive the vaccine, particularly the ability to see and spend time with loved ones and connect with social networks through family, friends, school, church, and work:

*[O]nce you have the vaccine*, *people will have more liberty*, *will want to get together in groups*, *and the first thing of all will be to see their family*, *their loved ones*, *right*? *Their friends*, *someone they’re close to*. *The feeling of hugging them that contact is what people are really yearning for*. Female, Latinx

#### Trusted messengers

Participants from all three racial-ethnic groups expressed trust in community-based organizations, including faith-based organizations and community-run clinics. For example, a Latina promotora (community health worker) who was interviewed remarked that “we are a trusted source to reach out to our community…the community identifies a lot with us.” Female, Latinx

Chinese American and Latinx participants were more likely than Black/African American participants to express confidence in health care and government institutions. In speaking about preferred places to receive a vaccination, one Chinese American participant stated:

*One is a place designated by the government*, *and the other is a large hospital*. *Because I think that the large hospitals must be approved or something by the government*. *I still believe in the government*. *I can accept it as long as the government approves it*. Female Chinese American

Chinese American participants mentioned that “the supervision of medical treatment and drugs is relatively strict [in the United States]” (Female, Chinese American) and that they had “confidence in the government and American medical care” (Female, Chinese American).

Several Latinx participants expressed trust in personal healthcare providers, such as *“*a medical expert who speaks Spanish, of course…who can give us a breakdown of what the vaccine contains.” For one Latinx individual, information from their personal doctor “would contribute a lot towards making my final decision.*”* (Female, Latinx)

### Access

#### Barriers

Participants from all three racial/ethnic groups experienced similar barriers to vaccine access during the early stages of the COVID-19 vaccine rollout. Participants expressed frustration with drive-in-only vaccination sites. Because of safety concerns, public transportation was not considered a viable option for travel. Limited locations and availability also prevented participants, especially elderly individuals, from accessing the vaccines:

*The elderly people around me have not gotten vaccinated because the sites of vaccination are too far away*. *I think enough supply and convenience should be the two factors that need to be taken into consideration if you want to improve the situation*. Female, Chinese American

Language barriers presented a barrier to access, especially for Latinx and Chinese. American participants with limited English proficiency. As one Chinese American participant explained, “if we go to a place where English is spoken, we will not understand. Not being able to communicate is a problem.” Female, Chinese American This problem was exacerbated when participants simultaneously experienced language barriers and difficulties navigating online appointment platforms and other technologies. One elderly participant explained that “we don’t read English, and we don’t know how to use the Internet well, so it’s difficult. It is really difficult for elderly people to make appointments and line up.” Female, Chinese American

#### Facilitators

When asked about facilitators to vaccine access, participants acknowledged that different communities had different needs. One individual suggested that “there should be different options, right? Because…we all have different ways of thinking and different needs.” Many participants expressed a desire for more community-run vaccination locations:

*I hope that our community center can provide vaccination*. *First*, *it is very close*. *I can go home and rest 15 minutes after the vaccination*. *I don’t need to take a bus*. *I think it’s best to get vaccinated in my community*. Female, Chinese American

Others suggested more outreach services to help individuals schedule an appointment and provide information about COVID-19 vaccines:

*It’s better for the doctor to call us and make an appointment for us*. *It’s impossible for us to make appointments by ourselves for the whole family to get vaccinated together*. *If the doctor makes an appointment*, *my family can get vaccinated together*. Female, Chinese American.

Participants emphasized that information should be presented in a way that the community could understand. Participants with limited English proficiency proposed language-concordant patient support, including outreach and on-site navigation:

*Where would [Spanish speakers] go to get the vaccine with staff that speaks Spanish, doctors that speak Spanish, nurses that speak Spanish? I think that would be a big help for people that don’t speak English*.

## Discussion

Our qualitative investigation in early 2021 of perspectives on vaccination among Black/African American, Latinx, and Chinese American community members in the San Francisco Bay area revealed several prominent themes during this early stage of the vaccine rollout, including information challenges and misinformation, mistrust of government and health institutions, and linguistic and other barriers to access. We found that vaccine misinformation from social media and other sources (e.g., family and friends) was common and powerful. So, too were knowledge gaps about vaccine safety, development, and efficacy. Misinformation and knowledge gaps interacted with longstanding justified skepticism among communities of color about the trustworthiness of health and government institutions rooted in systemic racism and anti-immigrant policies.

Our findings align with established conceptual frameworks for understanding vaccination behavior that emphasize how beliefs about benefits, harms, and trust may influence vaccine confidence, and as well as how barriers to vaccine access may affect vaccination behavior [33]. The themes that emerged from our study are consistent with results of surveys using quantitative methods that have found that issues of trust, experiences of racism, exposure to misinformation, and concerns about safety and efficacy are associated with COVID-19 vaccination intentions among Latinx and Black/African American populations [11–17,34]. Qualitative studies involving Black/African American and Latinx participants in other parts of the country have also reported similar concerns about the vaccine’s novelty, development process, and efficacy against variants [19–21,23–25,28].

While our findings accord with prior research in this area, they also deepen and expand understanding of the perspectives of members of the Black/African American, Latinx, and Chinese American communities about COVID-19 vaccination. Study participants frequently reported information gaps, exposure to vaccine misinformation, and social media as a source of vaccine information. Some expressed ambivalence about relying on social media to learn about vaccines, recognizing that the content often lacked scientific validity, but continuing to tune into these social media feeds [35]. Our study also revealed the problem of information overload, with participants struggling to discern take-home messages—a problem compounded not only by the rapidly evolving nature of the pandemic and public health guidelines but also by difficulty in finding information in languages other than English at the appropriate literacy level.

Many African American and Latinx participants cited structural and interpersonal racism, and anti-immigrant discrimination as factors reducing their trust in government and public health disseminated information and their willingness to be vaccinated [10,36,37]. Comments such as “You kept everything from us; now you suddenly want us first in line” highlight the potential for well-intentioned vaccine outreach efforts to communities of color to heighten vaccine skepticism if not designed and implemented with strong community input to earn trust. Although Chinese American participants less commonly voiced mistrust, participants from all groups emphasized the importance of local community- and faith-based organizations as trusted messengers of vaccine information.

Our study revealed the salience of social and health benefits as motivators for vaccination among a study population that included many individuals in low-wage “essential” occupations. Motivations to get vaccinated were shaped by experiences of economic and social hardship and loss among many participants, reflecting the disproportionate impact of COVID-19 in their communities. Participants’ strongest motivators were the need for personal and community economic stability, return to work and social connections, and protecting family and community members.

Our study also highlights the importance of addressing health system vaccine readiness in addition to individual readiness [9]. Participants mentioned a myriad of factors impeding access to vaccination in the early stages of vaccine rollout due to the emphasis on centralized mass vaccination sites using online scheduling, disadvantaging populations experiencing the “digital divide,” and limited transportation options. Language barriers were frequently cited as a concern. Although local government agencies produced translated vaccination materials, dissemination did not consistently reach the groups we studied.

Our study has important implications for strategies to promote equity in COVID-19 vaccination (Table 3). Outreach to Black/African American, Latinx, and Chinese American communities to enhance knowledge and address concerns about vaccination must recognize the structural racism that fuels mistrust in government and health institutions. Outreach should center and support trusted messengers who have visibility and deep roots in the community, especially individuals who are racially, culturally, and linguistically concordant with the population to be reached. Efforts must promote ongoing dialogue and not expect “one and done” marketing campaigns to be effective. Aligning messages and sharing data across health institutions is essential to driving a clear and concise message. Communication should address concerns and falsehoods and emphasize positive messages appealing to motivations to enhance community well-being and prosperity. Health systems must eliminate barriers contributing to vaccination inequity. Public health departments must invest in established community networks that know how to reach their populations, have the trained staff to communicate directly to residents and create a collaborative leadership model in working in and with the community. Without this kind of collaborative leadership model, we will continue to miss the mark.

**Table 3:** Study implications for vaccine outreach strategies.

**Vaccination Knowledge**		
Recognize Historical Underpinnings of Mistrust Center Trusted Messengers Emphasize Positive Messaging		Recognize historical underpinnings of mistrust of public health efforts and actively work to dismantle systemic racism in health care and research practices Center in outreach campaigns the voices and perspectives of trusted messengers with deep roots and visibility within the community (e.g., local celebrities, community figures, health care and faith leaders, athletes), with safe spaces and forums for ongoing dialogue and education Promote positive vaccination messaging about protecting family and community, reconnecting with friends and family, and safely returning to work.
**Trusted Messenger**		
Develop Aligned Messages Train Community Leaders Provide FAQs in multiple languages COVID-19 Vaccine Hotline		Pair trusted healthcare professionals and community leaders with local media and community-based organizations to develop aligned messages and responses to new developments. Train existing community peer leaders to implement on-the-ground outreach, answer vaccine concerns, and directly facilitate vaccine appointments for interested community members. Provide FAQs in multiple languages that can be disseminated on websites and flyers accessible to local communities. Provide a COVID vaccine hotline were trained, knowledgeable, culturally, and linguistically capable staff can answer individual questions and schedule vaccination for community members without internet access.
**Barriers and Facilitators**		
Vaccine Allocation to CBOs Outreach Scheduling Multi-lingual vaccination navigator Transportation	Allocate vaccines to community and faith-based organizations within impacted communities, staffed by trusted community leaders and workers. Conduct door to door outreach to assist with decision-making and scheduling; vaccinate in the household for homebound Staff multi-lingual phone hotlines, hold evening vaccination sessions, and reserve slots for drop-ins. Train and deploy multi-lingual vaccine navigators for door-to-door outreach, direct calls, and vaccination site assistance. Provide transportation to and from sites, including wheelchair-accessible vehicles.	

In an effort to bridge the gap between our recommendations and implementation, the study team produced a series of three 1-page *Practice and Policy Briefs* to rapidly disseminate preliminary study findings to stakeholders involved in vaccination efforts in the region. Stakeholders included local public health departments, CBO networks, and health care organizations. Local stakeholders pursued many of the strategies recommended in the Briefs, such as working in collaboration with key community leaders, establishing more vaccination hubs at community sites, problem solving with community leaders for COVID-19 mitigation, and acknowledging the structural racism and mistrust that exist in the local institutions. By the end of 2021, San Francisco had one of the highest vaccination rates in the US, with 85% of residents 5 years of age and older having received at least one vaccine dose and with the vaccination rates for Black/African American, Latinx, and Asian American populations equal to or higher than the rate for the white population [38].

Our study has several limitations. Study participants lived in the San Francisco area, and their views may not be generalizable to populations in other regions. Our study was conducted at a particular time in the pandemic when vaccines were just becoming available to limited public sectors; perspectives on COVID-19 vaccination may evolve based on new scientific information and community experience. For example, our study predated the Centers for Disease Control and Federal Drug Administration (FDA) pausing in April, 2020 the Janssen/Johnson and Johnson vaccine due to rare and sometimes fatal blood clotting in persons who received the this COVID-19 vaccine, which may have altered the perspectives and opinions of our participants subsequent to our study [39]. Qualitative methods cannot quantify the prevalence of beliefs, and our design was not structured to formally test for differences in beliefs across the three racial-ethnic groups studied.

In summary, this qualitative study of COVID-19 vaccine perspectives among Black/African American, Latinx, and Chinese American individuals provides insights into individual and health system readiness factors to inform strategies to redress vaccination inequities. Not only must vaccine outreach strategies include acknowledgment of how longstanding systemic, institutional, and structural racism contributes to mistrust in government and health institutions, it is also imperative to engage with, and support trusted messengers from the community to eliminate cultural, linguistic, and other barriers to vaccine access. As the US continues efforts to achieve comprehensive COVID-19 vaccination, including a new phase of implementing booster doses, vaccination strategies must prioritize reaching the racial and ethnic groups most adversely affected by COVID-19.

## Supporting information

S1 Table (DOC)
