# Supplementary material for: COVID-19 vaccination readiness among multiple racial and ethnic groups in the San Francisco Bay Area: A qualitative analysis
Source: PLoS One. 2022 May 12;17(5):e0266397. doi: 10.1371/journal.pone.0266397 (PMC9098010; doi:10.1371/journal.pone.0266397)
Supplement: S1 Table — (DOC) [file pone.0266397.s001.doc]

**S1. STOP COVID-19 CA Pre-questionnaire**

This is the pre-questionnaire to be completed for each participant before their participation in either the interview or focus group.

|  | STOP COVID Participant ID (see excel file on Box for numbering system) |  |
| --- | --- | --- |
|  |  |  |
|  | Was verbal consent obtained? | Yes  No (do not proceed) |
|  | Date consent obtained |  |
|  |  |  |
|  | Verbal consent obtained by |  |
|  |  |  |
|  | Which type of qualitative process is this for? | Interview Focus group |
|  | Name of individual administering this pre-questionnaire |  |
|  |  |  |
|  | Date of pre-questionnaire |  |
|  |  |  |
|  | **Pre- interview/focus group questionnaire** |  |
| 1 | What is the Zip Code where you live? |  |
|  |  |  |
| 2 | Are you currently working outside the home? | Yes No |
| 2a | (If yes) What is your occupation or type of job? |  |
|  |  |  |
| 3 | How many people live or stay in your household right now? Include yourself, any other adults, and any children |  |
| 3a | If >1 person (ie, anyone in addition to respondent): How many of these people are older than 64 years old? |  |
| 3b | If >1 person (ie, anyone in addition to respondent): How many of these people are less than 18 years old? |  |
| 4 | Do you or anyone else living in your household have a serious health condition like cancer, heart or lung disease, or a disease affecting the immune system? | Yes No |
| 5 | Did you receive an influenza flu shot in 2019 or 2020? | 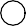 Yes 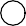 No |
| 6 | Have you ever been tested for COVID-19? | Yes No |

1. What is your gender or sex ?
2. How old are you? 18-40 years old
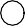
 41-64 years old 65 years old or older
3. With what racial or ethnic group or groups do you

identify (you can list more than one if you are multiracial or multiethnic)?


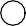

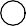


1. What is your preferred language for an interview or English group discussion for this study? Spanish

Cantonese Mandarin Another language


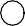

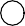

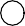

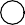

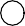


10a If another language, what other language?
